# TUG1 confers cisplatin resistance in esophageal squamous cell carcinoma by epigenetically suppressing PDCD4 expression via EZH2

**DOI:** 10.1186/s13578-018-0260-0

**Published:** 2018-11-28

**Authors:** Caihui Xu, Yinmou Guo, Haiyan Liu, Gongbin Chen, Yanju Yan, Teng Liu

**Affiliations:** 1 Department of Oncology, Shangqiu First People’s Hospital, No. 292 Kaixuan South Road, Shangqiu, 476100 China; 2 Department of Oncology, Xinxiang Medical College, No. 601 Jinsui Avenue, Hongqi District, Xinxiang, 453003 China

**Keywords:** Esophageal squamous cell carcinoma, Cisplatin, Taurine upregulated gene 1, Enhancer of zeste homolog 2, PDCD4

## Abstract

**Background:**

Increasing evidence has suggested the involvement of long non-coding RNA taurine upregulated gene 1 (TUG1) in chemoresistance of cancer treatment. However, its function and molecular mechanisms in esophageal squamous cell carcinoma (ESCC) chemoresistance are still not well elucidated. In the present study, we investigate the functional role of TUG1 in cisplatin (DDP) resistance of ESCC and discover the underlying molecular mechanism.

**Results:**

Our study revealed that TUG1 was up-regulated in DDP-resistant ESCC tissues and cells. High TUG1 expression was correlated with poor prognosis of ESCC patients. TUG1 knockdown improved the sensitivity of ECA109/DDP and EC9706/DDP cells to DDP. Moreover, TUG1 could epigenetically suppress PDCD4 expression via recruiting enhancer of zeste homolog 2. PDCD4 overexpression could mimic the functional role of down-regulated TUG1 in DDP resistance. PDCD4 knockdown counteracted the inductive effect of TUG1 inhibition on DDP sensitivity of ECA109/DDP and EC9706/DDP cells. Furthermore, TUG1 knockdown facilitated DDP sensitivity of DDP-resistant ESCC cells in vivo.

**Conclusion:**

TUG1 knockdown overcame DDP resistance of ESCC by epigenetically silencing PDCD4, providing a novel therapeutic target for ESCC.

**Electronic supplementary material:**

The online version of this article (10.1186/s13578-018-0260-0) contains supplementary material, which is available to authorized users.

## Background

Esophageal squamous cell carcinoma (ESCC) is one of the most frequent gastrointestinal malignancies in human, a sixth most common cause of cancer-related death worldwide [1, 2]. Despite the development of surgery combined with neoadjuvant radiation and/or chemotherapy, the majority of patients with ESCC were diagnosed frequently at the advanced stage and had poor prognosis [3, 4]. Chemoresistance frequently occurs during chemotherapy, which remains a major barrier to achieve successful treatment for ESCC [5, 6]. Therefore, it is urgent to elucidate the mechanism underlying chemoresistance in ESCC and develop novel therapeutic strategies to improve ESCC prognosis.

Long noncoding RNAs (lncRNAs) represent a class of endogenous non-protein-coding RNAs longer than 200 nucleotides [7]. Emerging evidence suggests the involvement of lncRNAs in normal development as well as tumorigenesis [8]. Dysregulated lncRNAs could act as oncogenic molecules and tumor suppressors in malignant tumors, closely associated with tumorigenesis, metastasis, diagnosis or prognosis [9]. Moreover, accumulating documents revealed that abnormal lncRNAs were related to chemotherapy resistance of cancers [10–12]. LncRNA taurine-upregulated gene 1 (TUG1), located on chromosome 22q12.2, was originally identified as a transcript up-regulated by taurine [13]. Recently, increasing evidence had suggested that aberrant TUG1 expression was associated with non-small cell lung cancer, hepatocellular carcinoma, and ESCC [14–16]. Although a previous study reported that high TUG1 expression was significantly correlated with chemotherapy resistance in ESCC, the function and mechanism of TUG1 in cisplatin (DDP) resistance of ESCC remains uncertain.

In this study, we aimed to investigate the expression and functional role of TUG1 in ESCC DDP resistance as well as its underlying molecular mechanism. Our study found that TUG1 expression was increased in ESCC tissues and cell lines, especially in DDP-resistant tissues and cells. Functionally, TUG1 knockdown improved the sensitivity of DDP-resistant ESCC cells to DDP. Mechanically, TUG1 improved the sensitivity of ESCC cells to DDP through epigenetically suppressing PDCD4 expression through recruiting enhancer of zeste homolog 2 (EZH2). Our study revealed a novel epigenetical regulatory mechanism between TUG1 and PDCD4 which could overcome DDP resistance in ESCC.

## Methods

### Sample collection and cell culture

The paired tumor tissues and adjacent normal tissues (n = 42) were collected from ESCC patients who underwent surgery resection at the Shangqiu first People’s Hospital. This study was approved by the Ethics Committee of Shangqiu first People’s Hospital and informed consents were signed by all patients. The normalized RNA-seq data of Esophageal Carcinoma (ESCA) were downloaded from the TCGA data portal website (https://cancergenome.nih.gov/).

Human immortalized esophageal epithelial cell line HET-1A and human ESCC cell lines (ECA109 and EC9706) were purchased from ATCC (Manassas, VA, USA). All cells were cultured in PRMI-1640 medium (Gibco, Rockville, MD, USA) supplemented with 10% FBS (Gibco) at 37 °C with 5% CO_2_. DDP-resistant variants (ECA109/DDP and EC9706/DDP) of ECA109 and EC9706 cells were established using a repetitive pulsatile treatment with constant concentrations of cisplatin [17]. The degree of chemotherapy resistance of DDP-resistant variants was evaluated before transfections.

### Cell transfection

Empty pcDNA3.1 vector (Vector) was obtained from Genepharma (Shanghai, China). TUG1 or PDCD4 overexpressing vector pcDNA3.1-TUG1 or pcDNA3.1-PDCD4 (TUG1 or PDCD4), small interfering RNAs against TUG1 (si-TUG1 #1 or si-TUG1 #2) or PDCD4 (si-PDCD4) and their scramble negative control (si-con) were chemically synthesized by Genepharma (Shanghai, China). All cell transfections were performed using the Lipofectamine 2000 (Invitrogen, Carlsbad, CA, USA).

### Quantitative real-time PCR (qRT-PCR)

Total RNA was isolated from ESCC tissues and cells using Trizol reagent (Invitrogen) and then reversely transcribed into cDNA using PrimeScript RT Reagent Kit (TaKaRa, Dalian, China). TUG1 and PDCD4 expression levels were detected by quantitative real-time PCR with SYBR Green Master Mix (TOYOBO, Osaka, Japan) using an Applied Biosystems 7500 Real-Time PCR Systems (Applied Biosystems, Foster City, CA, USA). The primes were as follows: TUG1 forward, 5′-TAGCAGTTCCCCAATCCTTG-3′, TUG1 reverse, 5′-CACAAATTCCCATCATTC CC-3′; PDCD4 forward, 5′-GGCCTCCAAGGAGTAAGACC-3′, PDCD4 reverse, 5′-AGGGGTCTACATGGCAACTG-3′. Data were analyzed using the comparative Ct method (2^−ΔΔCt^) with GAPDH as an internal control.

### Drug sensitivity assay

The cell viability of ECA109/DDP and EC9706/DDP cells and their parental cells was measured by 3-(4,5-dimethyl-2-thiazolyl)-2,5-diphenyltetrazolium bromide (MTT) (Sigma, St. Louis, Missouri, USA) assay. DDP sensitivity was determined using the IC50 value (half maximal inhibitory concentration).

### Flow cytometric analysis

Cell apoptosis was evaluated using Annexin V-FITC/PI Apoptosis Detection Kit (KeyGEN Biotech, Nanjing, China) as described previously [18]. Briefly, ECA109/DDP and EC9706/DDP cells with different transfection were treated with 20 μM DDP for 48 h, followed by double stained with Annexin V-FITC and PI under a dark condition. Cell apoptotic rates were evaluated by FACSan flow cytometry (BD Biosciences, San Jose, CA, USA).

### Subcellular fraction assays

The separation of the nuclear and cytosolic fractions of ECA109 cells was performed using the PARIS Kit (Life Technologies, Carlsbad, CA, USA) following the manufacturer’s instructions.

### RNA pull-down assays

TUG1 and anti-sense-TUG1 was transcribed with TranscriptAid T7 High Yield Transcription Kit (Thermo Fisher Scientific) and then labeled with Thermo Scientific Pierce RNA 3′ Desthiobiotinylation Kit (Thermo Fisher Scientific). Pierce Magnetic RNA-Protein Pull down Kit (Thermo Fisher Scientific) was used to perform RNA pull down assay. Briefly, labeled RNAs were bound with Streptavidin Magnetic Beads and then incubated with ECA109/DDP cell protein lysates. Then the RNA-binding proteins were eluted for the further western blot analysis.

### RNA immunoprecipitation (RIP) assays

RIP experiments were performed using Magna RIP™ RNA-Binding Protein Immunoprecipitation Kit (Millipore, Bedford, MA, USA) according to the manufacturer’s protocol. EZH2 and IgG antibodies were obtained from Cell Signaling Technology (Danvers, MA, USA). The co-precipitated RNAs were purified and analyzed by qRT-PCR analysis.

### Chromatin immunoprecipitation (ChIP) assays

Chromatin immunoprecipitation assay was performed to confirm the interaction between TUG1 and PDCD4 gene using EZ-ChIP kit (Millipore). The chromatins were immunoprecipitated with antibodies against EZH2 (Cell Signaling Technology), H3K27me3 (Millipore) or IgG (Millipore). Finally, the immunoprecipitated chromatin was purified and analyzed by qRT-PCR analysis. Primers for PDCD4 promoter region were 5′-GGTCTGGGAAGCTCCGATTT-3′ (forward) and 5′-GCAGTTGGTGGTCATCCTCA-3′ (reverse).

### Luciferase reporter assay

PDCD4 promoter sequences were inserted into pGL3-Basic luciferase plasmid (Promega, Madison, WI, USA) to generate PDCD4 promoter reporter vector. Then, PDCD4 promoter reporter was transfected into ECA109/DDP cells using Lipofectamine 2000 (Invitrogen) along with phRL-TK vector (Promega) and (Vector or TUG1) or (si-con or si-TUG1). Luciferase Reporter assay system (Promega) was performed to detect luciferase activity in ECA109/DDP cells 48 h post-transfection.

### Western blot analysis

Western blotting was performed according to our previously reported protocol [19]. The primary antibodies anti-EZH2, anti-PDCD4 and anti-GAPDH were obtained from Cell Signaling Technology (Danvers, MA, USA).

### Animal experiments

The animal experiment was performed according to the national standard of the care and use of laboratory animals and got the approval of the Ethics Committee of Shangqiu first People’s Hospital. ECA109/DDP cells were infected with sh-TUG1 or sh-con lentivirus, followed by the sieving using puromycin (Sigma-Aldrich, St. Louis, MO, USA) for nearly 7 days to construct stable lentivirus-transfected ECA109/DDP cell line. Then, ECA109/DDP cells (1.0 × 10^7^) stably infected with sh-TUG1 or sh-con were subcutaneously injected into the tail veins of BALB/c-nude mice (4 weeks old) from the Shanghai Experimental Animal Center of the Chinese Academy of Sciences (Shanghai, China). One week later, mice were intraperitoneally injected with 6 mg/kg DDP or same volume of PBS every week according to indicated groups (n = 5 each group): sh-con + PBS, sh-TUG1 + PBS, sh-con + DDP, sh-TUG1 + DDP. The tumor sizes were measured every week. After 42 days, the mice were killed, and the tumor weights were detected. qRT-PCR and western blot assays were performed to detect TUG1 expression and PDCD4 protein levels.

### Statistical analysis

All data were presented as means ± standard deviation (SD). Student’s t-test and one-way ANOVA were used to calculate the statistic difference using SPSS 16.0 software (SPSS, Inc., Chicago, IL, USA). Differences were considered statistically significant when *P* value < 0.05.

## Results

### TUG1 was increased in DDP-resistant ESCC tissues and cells

**Fig. 1:**
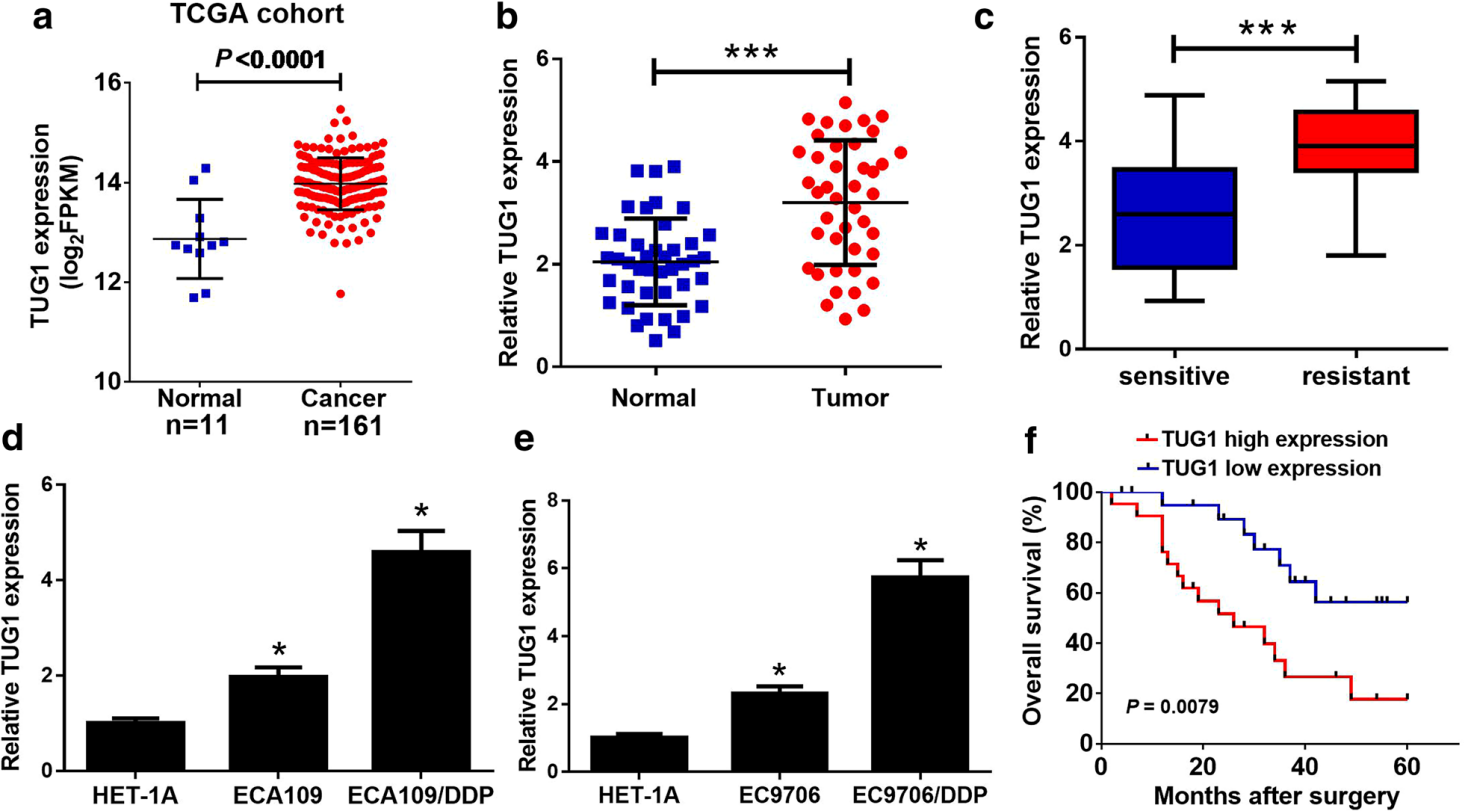
TUG1 was up-regulated in DDP-resistant ESCC tissues and cell lines. qRT-PCR analysis indicated the TUG1 expression levels in ESCC tumor or normal tissues from TCGA dataset (**a**), ESCC tumor or adjacent normal tissues (n = 42) (**b**), DDP-sensitive or DDP-resistant ESCC tissues (**c**), and DDP-resistant ESCC cell lines (ECA109/DDP and EC9706/DDP) and their parental cells (ECA109 and EC9706) or normal immortalized esophageal epithelial cell line HET-1A (**d**, **e**). **f** The overall survival was evaluated by Kaplan–Meier curve between low and high TUG1 expression groups. *P < 0.05

To explore the role of TUG1 in ESCC, we firstly detected the expression of TUG1 in ESCC tissues from TCGA databases. TUG1 expression was significantly elevated in ESCC tumor tissues compared with normal tissues (Fig. 1a). To confirm the result, we further investigated TUG1 expression in ESCC tumor tissues and adjacent normal tissues by qRT-PCR analysis. Consistently, TUG1 was significantly up-regulated in ESCC tissues compared with adjacent normal tissues (Fig. 1b). Additionally, compared with DDP-sensitive (n = 21) ESCC tissues, TUG1 expression was strangely increased in DDP-resistant (n = 21) ESCC tissues (Fig. 1c). To further confirm the expression level of TUG1 in ESCC cells, qRT-PCR analysis was performed in ESCC parental cell lines (ECA109 and EC9706), DDP-resistant cell lines (ECA109/DDP and EC9706/DDP) and normal immortalized esophageal epithelial cell line HET-1A. The results showed that expression of TUG1 was dramatically improved in ECA109 and EC9706 cells compared with HET-1A cells (Fig. 1d, e). Particularly, ECA109/DDP and EC9706/DDP cells exhibited higher TUG1 level than their parental cells (Fig. 1d, e). Kaplan–Meier survival analysis revealed that patients with high TUG1 level (n = 21) had a low overall survival (*P* = 0.0079) compared with that with low TUG1 level (n = 21) (Fig. 1f). All these data demonstrated that up-regulation of TUG1 may be implicated with DDP resistance in ESCC.

### TUG1 knockdown overcame DDP resistance of ESCC cells

**Fig. 2:**
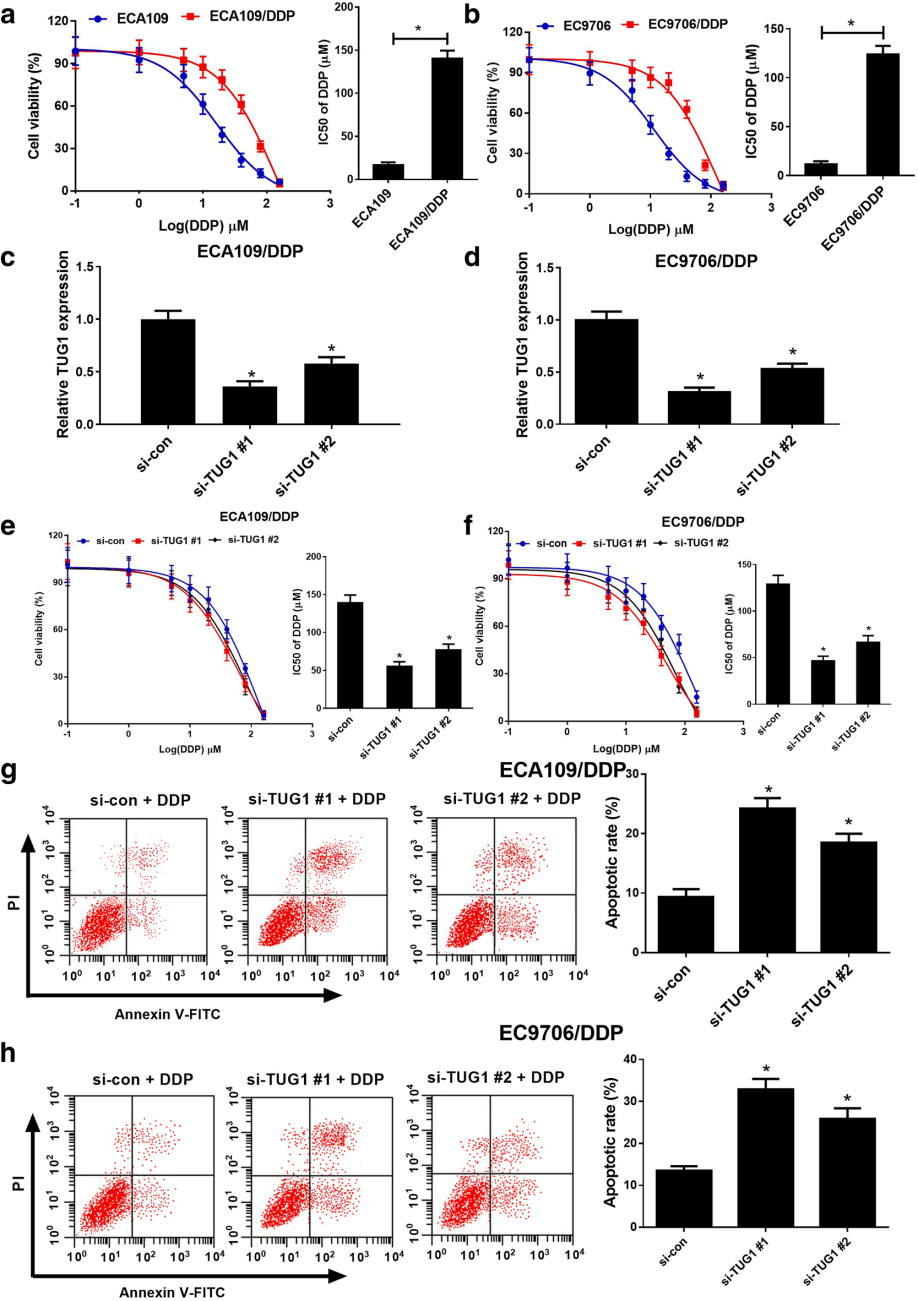
TUG1 knockdown overcame DDP resistance of ESCC cells. **a**, **b** The cell viability was determined by MTT assay in ECA109/DDP and EC9706/DDP cells and their parental cells exposed to different concentrations of DDP (0.1, 1, 5, 10, 20, 40, 80, 160 μM) for 48 h. **c**, **d** qRT-PCR analysis was performed in ECA109/DDP and EC9706/DDP cells transfected with TUG1 siRNAs (si-TUG1 #1 or si-TUG1 #2) or si-con. **e**, **f** ECA109/DDP and EC9706/DDP cells transfected with siRNAs (si-TUG1 #1 or si-TUG1 #2) or si-con were treated with various concentrations of DDP (0.1, 1, 5, 10, 20, 40, 80, 160 μM) for 48 h and cell viability was evaluated by MTT assay. **g**, **h** Cell apoptosis was determined by flow cytometry analysis in siRNAs (si-TUG1 #1 or si-TUG1 #2) or si-con transfected ECA109/DDP and EC9706/DDP cells after treatment with 20 μM of DDP. *P < 0.05

To evaluate the DDP resistance of ECA109/DDP and EC9706/DDP cells, IC50 of DDP was determined by MTT assay in ECA109/DDP and EC9706/DDP cells and their parental cells. Compared with their parental cells, ECA109/DDP and EC9706/DDP cells presented poor response to DDP, as evidenced by the increased IC50 (Fig. 2a, b). To further investigate the role of TUG1 in DDP-resistant ESCC cells, TUG1 siRNAs (si-TUG1 #1 or si-TUG1 #2) or si-con were transfected into ECA109/DDP and EC9706/DDP cells. qRT-PCR analysis discovered that TUG1 siRNAs demonstrably weakened TUG1 expression in ECA109/DDP and EC9706/DDP cells (Fig. 2c, d). Remarkably, TUG1 knockdown heightened the sensitivity of ECA109/DDP and EC9706/DDP cells to DDP (Fig. 2e, f). To further determine the effect of TUG1 on DDP-induced apoptosis, flow cytometry analysis was carried out in ECA109/DDP and EC9706/DDP cells exposed to 20 μM DDP. As expected, TUG1 exhaustion remarkably improved DDP-induced apoptosis in ECA109/DDP and EC9706/DDP cells (Fig. 2g, h). Together, silencing of TUG1 enhanced DDP sensitivity in ESCC cells.

### TUG1 epigenetically suppressed PDCD4 expression in ESCC cells

**Fig. 3:**
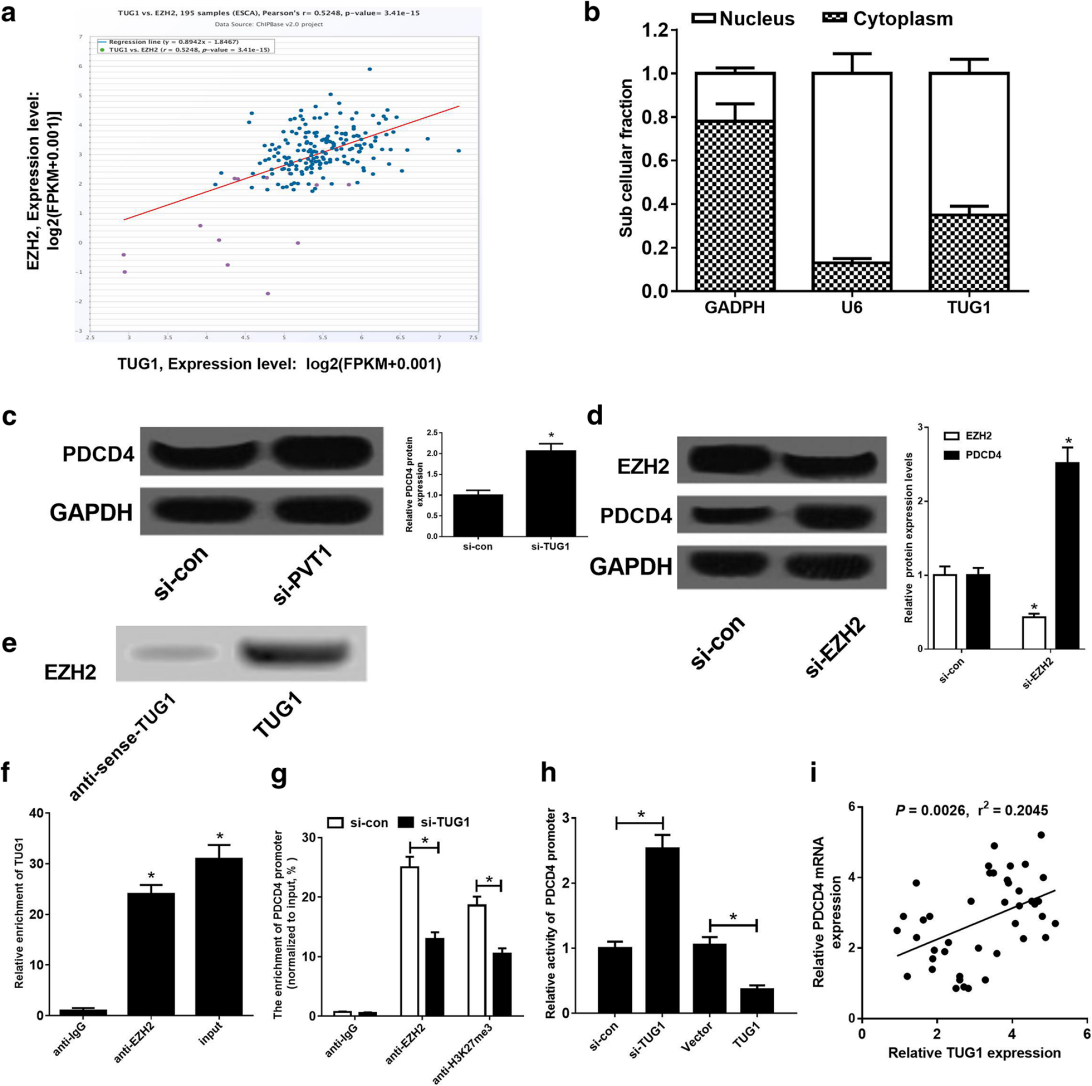
TUG1 epigenetically silenced PDCD4 expression by recruiting EZH2 in ESCC cells. **a** Pearson correlation analysis between TUG1 and EZH2 in 195 tumor tissue samples of ESCC from TCGA. **b** TUG1 subcellular location was explored in EC9706/DDP cells. GAPDH acted as a cytoplasm control and U6 used as a nucleus control. **c** PDCD4 protein levels in ECA109/DDP cells transfected with si-con or si-TUG1. **d** EZH2 and PDCD4 protein levels in ECA109/DDP cells transfected with si-con or si-EZH2. **e** RNA pull-down assay indicated the binding of TUG1 with EZH2. **f** RIP assay was performed in ECA109/DDP cells and the co-precipitated RNA was subjected to qRT-PCR for TUG1. **g** qRT-PCR analysis following ChIP was performed to evaluate the enrichment of EZH2 and H3K27me3 on the PDCD4 promoter in ECA109/DDP cells. **h** Luciferase reporter assay was performed to evaluate the activity of the PDCD4 promoter in ECA109/DDP cells transfected with (si-con or si-TUG1) or (Vector or PDCD4). **i** The correlation analysis between TUG1 and PDCD4 expression in ESCC tumor specimen. *P < 0.05

Previous studies demonstrated that TUG1 could regulate gene expression by recruiting EZH2, leading to the increase of H3K27me3 level in promoter regions of target genes [20, 21]. Moreover, PDCD4 as a tumor suppressor was previously reported to be suppressed by PRC2 complex via increasing the level of H3K27me3 at its promoter in glioma [22]. Hence, we further investigated whether TUG1 epigenetically suppressed PDCD4 in DDP-resistant ESCC cells by recruiting EZH2. Firstly, Chipbase database (http://rna.sysu.edu.cn/chipbase/) was used to analyze the correlation between TUG1 and EZH2 in 195 ESCC tissue samples from TCGA databases. The results showed that TUG1 expression was positively associated with EZH2 level in ESCC tissue samples (Fig. 3a). qRT-PCR analysis revealed that TUG1 was mainly located in nucleus of ECA109/DDP cells (Fig. 3b), suggesting that TUG1 may exert its regulatory role at the transcriptional level. Western blot analysis showed that TUG1 knockdown significantly raised PDCD4 expression in ECA109/DDP cells (Fig. 3c). Moreover, si-EZH2 transfection suppressed EZH2 and up-regulated PDCD4 expression in ECA109/DDP cells (Fig. 3d). To confirm whether TUG1 directly bound to EZH2 in ECA109/DDP cells, RNA pull-down and RIP assays were performed in ECA109/DDP cells. As expected, TUG1 could pull down EZH2 protein (Fig. 3e) and TUG1 was noticeably enriched by EZH2 antibody (Fig. 3f). All these data demonstrated that TUG1 could directly interact with EZH2 in ESCC cells. To further confirm whether TUG1 transcriptionally repressed PDCD4 expression through enrichment of EZH2 and H3K27me3 on the PDCD4 promoter, ChIP assays were performed in ECA109/DDP cells. The results revealed that TUG1 knockdown dramatically decreased EZH2 recruitment and H3K27me3 levels on the PDCD4 promoter in ECA109/DDP cells (Fig. 3g). The transcription suppressive effect of TUG1 was further verified by luciferase reporter assay. As expected, TUG1 knockdown could enhance the activity of PDCD4 promoter, oppositely, TUG1 overexpression led to reduction of the promoter activity (Fig. 3h). Moreover, PDCD4 expression was positively correlated with TUG1 expression (Fig. 3i) in ESCC tumor specimens. All these data suggested that TUG1 epigenetically suppressed PDCD4 expression by recruiting EZH2 to the promoter region of PDCD4 and increasing H3K27me3 level in ESCC cells.

### PDCD4 overexpression enhanced DDP sensitivity of ESCC cells

**Fig. 4:**
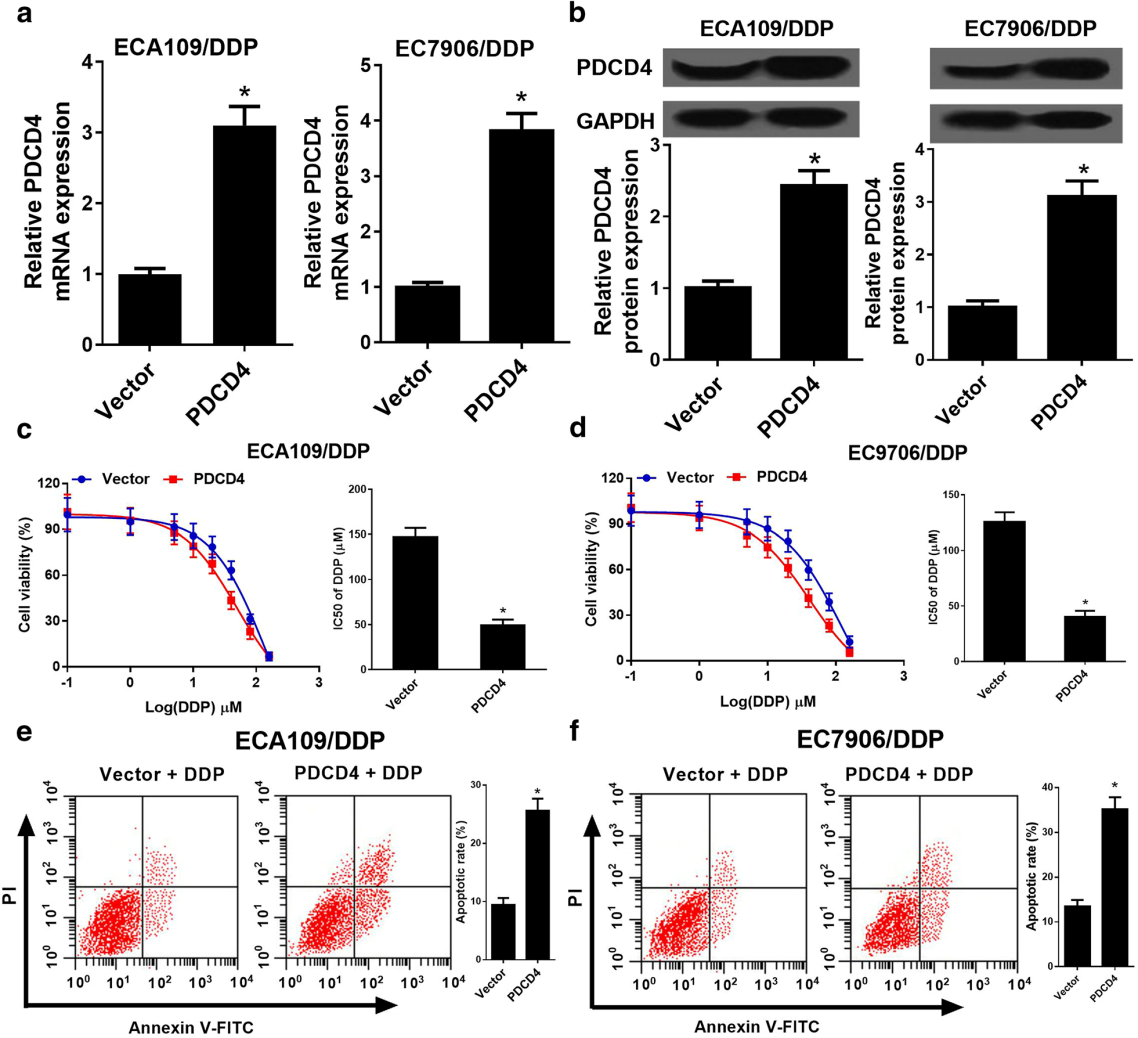
PDCD4 overexpression improved DDP sensitivity of ESCC cells. ECA109/DDP and EC9706/DDP cells were transfected with Vector or PDCD4, followed by determination of PDCD4 expression by qRT-PCR and western blot analyses (**a**, **b**), IC50 of DDP by MTT assay (**c**, **d**), and cell apoptosis by flow cytometry analysis (**e**, **f**). *P < 0.05

To further investigate the role of PDCD4 in DDP-resistant ESCC cells, PDCD4 overexpressing vector (PDCD4) or empty vector (Vector) was transfected into ECA109/DDP and EC9706/DDP cells. qRT-PCR and western blot analyses indicated that PDCD4 mRNA and protein expression levels were dramatically increased in PDCD4 transfected ECA109/DDP and EC9706/DDP cells (Fig. 4a, b). Moreover, PDCD4 overexpression improved the DDP sensitivity (Fig. 4c, d) and inhibited cell proliferation capacity (Additional file 1: Figure S1A, B) of ECA109/DDP and EC9706/DDP cells. To further determine the effect of PDCD4 on DDP-induced apoptosis, flow cytometry analysis was performed in ECA109/DDP and EC9706/DDP cells treatment with 20 μM DDP. As expected, PDCD4 overexpression extremely boosted DDP-induced apoptosis in ECA109/DDP and EC9706/DDP cells (Fig. 4e, f). Moreover, PDCD4 overexpression could also promote apoptosis of ECA109/DDP and EC9706/DDP cells (Additional file 1: Figure S1C, D). Together, up-regulation of PDCD4 improved DDP sensitivity in ESCC cells.

### TUG1 knockdown facilitated DDP sensitivity of ESCC cells through increasing PDCD4 expression

**Fig. 5:**
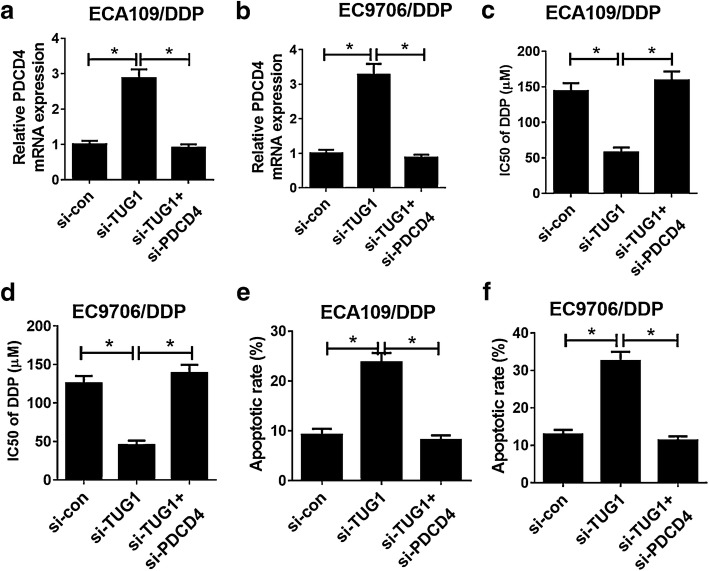
PDCD4 knockdown reversed the enhancive effect of down-regulated TUG1 on DDP sensitivity of ESCC cells. ECA109/DDP and EC9706/DDP cells were transfected with si-con, si-TUG1 or si-TUG1 + PDCD4, followed by determination of PDCD4 expression by qRT-PCR analysis (**a**, **b**), IC50 of DDP by MTT assay (**c**, **d**), and cell apoptosis by flow cytometry analysis (**e**, **f**). *P < 0.05

To further study whether TUG1 exerted its functional role in DDP resistance of ESCC cells through regulating PDCD4 expression, ECA109/DDP and EC9706/DDP cells were transfected with si-con, si-TUG1 or si-TUG1 + si-PDCD4. qRT-PCR analysis revealed that TUG1 inhibition increased PDCD4 expression in ECA109/DDP and EC9706/DDP cells, which was particularly reversed by PDCD4 knockdown (Fig. 5a, b). MTT assay revealed that down-regulation of TUG1 improved DDP sensitivity of ECA109/DDP and EC9706/DDP cells, nevertheless, the inductive effect of TUG1 inhibition on DDP sensitivity was strikingly eliminated by PDCD4 silencing (Fig. 5c, d). Furthermore, introduction of si-PDCD4 particularly demolished the inductive effect of down-regulated TUG1 on apoptosis in ECA109/DDP and EC9706/DDP cells (Fig. 5e, f). Collectively, these results confirmed that TUG1 knockdown facilitated DDP sensitivity of ESCC cells through elevating PDCD4 expression.

### TUG1 knockdown enhanced DDP sensitivity in tumors in vivo

**Fig. 6:**
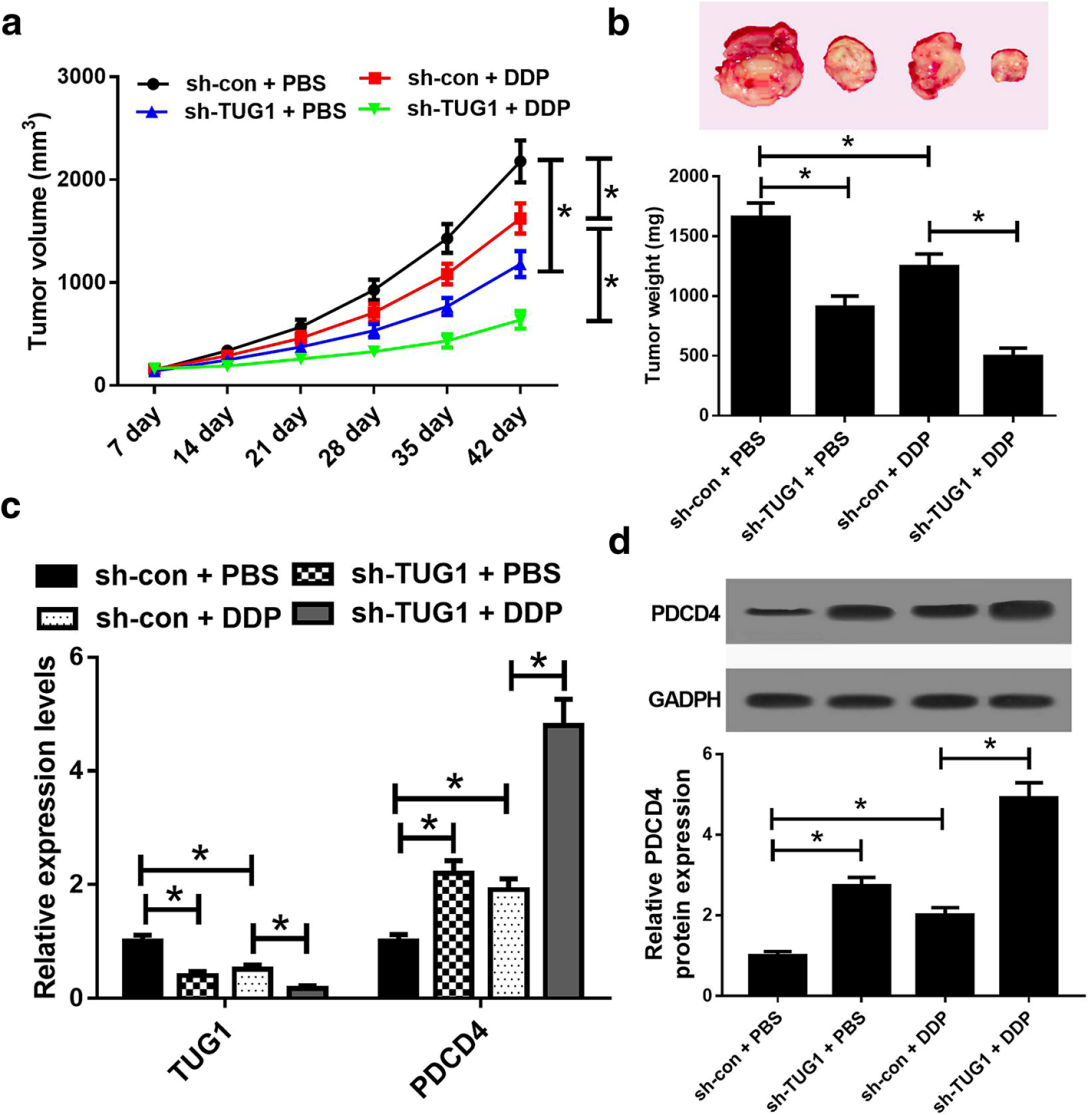
TUG1 knockdown sensitized ESCC cells to DDP in vivo. ECA109/DDP cells stably transfected with sh-con or sh-TUG1 were subcutaneously inoculated into the nude mice, followed by treatment with PBS or DDP. Mice were killed and removed tumors at 42 days after inoculation. **a** The tumor volumes were measured every week. **b** The typical photographs and average weights of resected tumors. **c** qRT-PCR analysis of TUG1 and PDCD4 mRNA levels in excised tumor tissues. **d** Western blot analysis PDCD4 protein level in excised tumor tissues. *P < 0.05

To further confirm the functional role of TUG1 in DDP resistance in vivo, ECA109/DDP cells infected with sh-con or sh-TUG1 were subcutaneously injected into the nude mice to generate xenograft, followed by treatment with DDP or PBS. The data revealed that TUG1 knockdown or DDP treatment significantly suppressed tumor growth, evidenced by the diminished tumor volume (Fig. 6a) and tumor weight (Fig. 6b). Moreover, TUG1 knockdown combined with DDP treatment led to a more distinct reduction on tumor growth, suggesting down-regulation of TUG1 enhanced the DDP sensitivity of ESCC cells in vivo (Fig. 6a, b). Additionally, qRT-PCR assay revealed that TUG1 mRNA levels were lowered, while PDCD4 expression was elevated in tumors after sh-TUG1 introduction or DDP treatment (Fig. 6c), especially after combination of sh-TUG1 introduction and DDP treatment. Western blot analysis revealed that TUG1 knockdown or DDP exposure pointedly increased PDCD4 protein level in tumor tissues (Fig. 6d). The combination of TUG1 knockdown and DDP exposure led to much higher PDCD4 protein expression (Fig. 6d). All these data proved that TUG1 knockdown improved DDP sensitivity of ESCC cells in vivo.

## Discussion

Acquiring chemoresistance have restricted treatment outcome for ESCC patients in the clinic. Hence, it is essential to investigate the molecular mechanism underlying chemoresistance and identify novel targets for chemoresistance therapy. In this study, we found that the expression level of TUG1 was significantly elevated in DDP-resistant ESCC tissues and cells. Moreover, TUG1 knockdown re-sensitized ECA109/DDP and EC9706/DDP cells to DDP by promoting DDP-induced apoptosis. More importantly, TUG1 conferred DPP resistance to ESCC cells via epigenetically silencing PDCD4 via EZH2. Therefore, TUG1 may be a promising therapeutic target for DDP resistance in ESCC.

Elucidating the molecular mechanism underlying chemoresistance could contribute to develop reasonable and effective therapies to overcome chemoresistance. Our results demonstrated that TUG1 expression level was elevated in DDP-resistant ESCC tissues and cells, and down-regulation of TUG1 re-sensitized ECA109/DDP and EC9706/DDP cells to DDP. Apart from our findings, dysregulated TUG1 has been reported to be implicated with chemoresistance in other cancers. For example, TUG1 was overexpressed in small cell lung cancer, and TUG1 down-regulation sensitized lung cancer cells to chemotherapeutic drugs (DDP, Adriamycin and Etoposide) by epigenetically suppressing LIM-kinase 2b (LIMK2b) expression through EZH2 [21]. Moreover, TUG1 knockdown re-sensitized MTX-resistant colorectal cell lines to MTX through acting as a competitive endogenous RNA (ceRNA) to sponge miR-186 and release the miRNA target CPEB2 [23]. On the contrary, TUG1 expression was down-regulated in triple negative breast cancer, and overexpression of TUG1 enhance DDP sensitivity in MDA-MB-231 and BT549 cells by sponging miR-197 [24]. All these findings suggested that the TUG1 could be used as a promising therapeutic target for chemoresistance in cancers.

The precise mechanism by which TUG1 up-regulation contributed to DDP resistance in ESCC was unclear. Hence, the functional mechanism of TUG1 was further investigated in the present study. Previous studies found that about 20% of lncRNAs can bind to polycomb repressive complex 2 (PRC2), which subsequently induced the silence of targeted genes through harboring methyltransferase activity [25]. Moreover, TUG1 has been proved to regulate genes expression by binding with EZH2 in human non-small cell lung cancer, gastric cancer and hepatocellular carcinoma [14, 26, 27]. EZH2, a vital catalytic subunit of PRC2, is a histone methyltransferase that epigenetically represses gene expression by promoting histone H3 lysine 27 trimethylation (H3 K27me3) [28, 29]. PDCD4, a tumor suppressor, was recently demonstrated to be negatively regulated by CASC15, via recruiting EZH2 and subsequently changing H3 K27me3 level in melanoma [30]. Therefore, we further investigated whether TUG1 could regulate PDCD4 expression by recruiting EZH2. Our western blot assays indicated that TUG1 or EZH2 knockdown elevated PDCD4 protein levels. Moreover, RNA pull-down and RIP assays further validated that TUG1 could bind to EZH2. ChIP and luciferase reporter assays further proved that TUG1 knockdown enhanced the promoter activity of PDCD4 by attenuating the recruiting of EZH2 on PDCD4 promoter region. These data demonstrated that TUG1 epigenetically silencing PDCD4 via recruiting EZH2 in ECA109/DDP cells. PDCD4 has been identified as a tumor suppressor in multiple cancers [31, 32]. Moreover, PDCD4 could improve the sensitivity of cancer cells to chemotherapy drugs such as docetaxel and cisplatin [33, 34]. Particularly, overexpression of PDCD4 induced apoptosis and enhanced chemosensitivity to cisplatin in ESCC [35]. Consistently, our data also revealed that PDCD4 overexpression could overcome DDP resistance in ECA109/DDP and EC9706/DDP cells. Furthermore, PDCD4 inhibition reversed the inductive effect of TUG1 knockdown on the sensitivity of ECA109/DDP and EC9706/DDP to DDP. All these data demonstrated that TUG1 inhibition sensitized DDP-resistant ESCC cells to DDP through epigenetically silencing PDCD4 in ESCC.

## Conclusions

In conclusion, our study demonstrated that TUG1 knockdown enhanced DDP sensitivity of ESCC cells. Importantly, the enhancive effect of TUG1 inhibition on DDP sensitivity might be mediated by PDCD4 through an epigenetic mechanism in ESCC cells, providing a promising therapeutic strategy to overcome DDP resistance in ESCC.

## Additional file

**Additional file 1: Figure S1.** PDCD4 overexpression suppressed proliferation and induced apoptosis of ESCC cells. ECA109/DDP and EC9706/DDP cells were transfected with Vector or PDCD4, followed by determination of cell proliferation by MTT assay (A and B), and cell apoptosis by flow cytometry analysis (C and D). *P < 0.05.
