# Supplementary material for: TUG1 confers cisplatin resistance in esophageal squamous cell carcinoma by epigenetically suppressing PDCD4 expression via EZH2
Source: Cell Biosci. 2018 Nov 28;8:61. doi: 10.1186/s13578-018-0260-0 (PMC6263046; doi:10.1186/s13578-018-0260-0)
Supplement: Supplementary file 1 — Additional file 1: Figure S1. PDCD4 overexpression suppressed proliferation and induced apoptosis of ESCC cells. ECA109/DDP and EC9706/DDP cells were transfected with Vector or PDCD4, followed by determination of cell proliferation by MTT assay (A and B), and cell apoptosis by flow cytometry analysis (C and D). *P < 0.05. [file 13578_2018_260_MOESM1_ESM.docx]

**
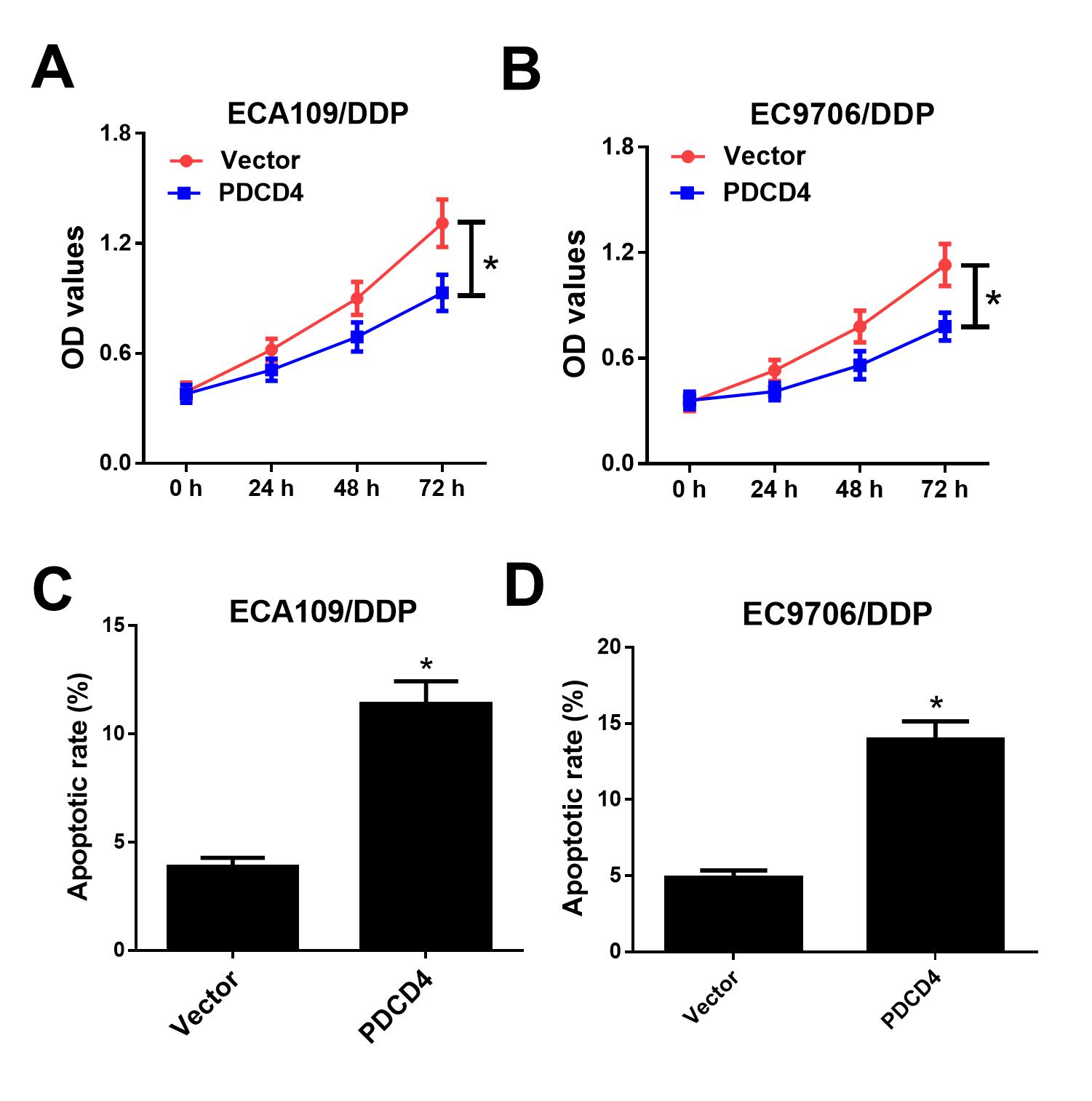
**

**Figure S1.** **PDCD4 overexpression suppressed proliferation and induced apoptosis of ESCC cells**. ECA109/DDP and EC9706/DDP cells were transfected with Vector or PDCD4, followed by determination of cell proliferation by MTT assay (A and B), and cell apoptosis by flow cytometry analysis (C and D). **P* < 0.05.
